# From Conservative Management to Surgical Rescue: A Case of Emphysematous Pyelonephritis in Uncontrolled Diabetes

**DOI:** 10.7759/cureus.109512

**Published:** 2026-05-23

**Authors:** Efrah Ashraf, Arshia Ahmed, Muhammad A Tariq, Sumreen Nazly, Shannay Bellamy

**Affiliations:** 1 Internal Medicine, Guthrie Lourdes Hospital, Binghamton, USA; 2 Internal Medicine, Guthrie Lourdes Hospital, Binghamton , USA; 3 Medical College, University Medical and Dental College, Faisalabad, PAK

**Keywords:** against medical advice discharge, diabetes mellitus, emphysematous pyelonephritis, nephrectomy, patient compliance, percutaneous drainage

## Abstract

Emphysematous pyelonephritis (EPN) is a severe necrotizing renal infection occurring predominantly in patients with diabetes mellitus. Modern management favors a stepwise approach with medical therapy and percutaneous drainage, reserving nephrectomy for refractory cases. However, early identification of high-risk features remains critical to optimizing outcomes.

We are highlighting the case of a 53-year-old female with no prior medical care who presented with left flank pain and was found to have severe EPN with multiple renal abscesses, newly diagnosed diabetes mellitus (glycated hemoglobin (HbA1c) 14%), and stage 5 chronic kidney disease (glomerular filtration rate (GFR) 12 mL/min/1.73 m²). Despite initial percutaneous drainage and intravenous antibiotics, she left the hospital prematurely against medical advice and was discharged on oral antibiotics. She re-presented 13 days later with worsening infection requiring a second drainage procedure. Due to persistent clinical deterioration despite repeated interventions, she ultimately underwent successful robotic left radical nephrectomy with complete recovery.

This case illustrates the challenges of managing EPN when complicated by extreme metabolic derangement, severe renal dysfunction, and patient non-compliance. It demonstrates that while conservative management is preferred, early recognition of high-risk features and timely escalation to nephrectomy are essential when conservative measures fail. The interrupted treatment course highlights real-world barriers to optimal care and the importance of patient education and shared decision-making.

## Introduction

Emphysematous pyelonephritis (EPN) is an acute, severe necrotizing infection of the renal parenchyma and surrounding tissues characterized by gas formation within the kidney, collecting system, or perinephric space [1]. This rare but life-threatening condition occurs predominantly in patients with diabetes mellitus, affecting up to 95% of cases, and carries a mortality rate of 13%-40% depending on disease severity and management approach [1-3]. The pathogenesis involves gas-forming organisms, most commonly Escherichia coli (70% of cases), in the setting of impaired host immunity and tissue ischemia associated with poorly controlled diabetes [1,4].

The management of EPN has evolved significantly over the past two decades. While emergency nephrectomy was historically the standard treatment, contemporary evidence supports a stepwise approach prioritizing medical management with broad-spectrum antibiotics and percutaneous drainage (PCD), reserving nephrectomy for cases that fail conservative therapy [2,5]. This paradigm shift reduced mortality from 50% with medical management alone and 25% with emergency nephrectomy to 13.5% with combined medical management and PCD [2]. However, certain high-risk features, including severe renal dysfunction, extensive disease (Huang classification ≥3B), thrombocytopenia, and septic shock, predict conservative management failure and may necessitate earlier surgical intervention [4,5].

Despite advances in treatment protocols, patient-related factors such as compliance with therapy and premature discharge remain underreported challenges that can significantly impact outcomes. We present a case of EPN in a patient with newly diagnosed diabetes and extreme metabolic derangement whose treatment course was complicated by premature self-discharge, subsequent treatment failure, and ultimate requirement for nephrectomy. This case highlights the real-world complexities of managing severe infections when optimal care is interrupted by patient non-compliance and illustrates the importance of early risk stratification and timely surgical escalation.

## Case presentation

A 53-year-old female with no known past medical history presented to the emergency department with a two-week history of severe left flank pain radiating to the back, accompanied by decreased urine output. The patient reported that she does not routinely seek medical care and had not seen a physician for several years. She denied associated chills, nausea, or vomiting. There was no history of recent travel, immobilization, or trauma.

On presentation, vital signs revealed mild tachycardia with a heart rate of 108 beats per minute; other vital signs were within normal limits. Physical examination demonstrated abdominal distention with preserved bowel sounds and bilateral costovertebral angle tenderness without guarding or rebound tenderness. Cardiovascular and pulmonary examinations were unremarkable.

On presentation, laboratory evaluation revealed significant abnormalities (Table 1). Notable findings included marked leukocytosis (white blood cell count 18.85 × 10³/µL; reference range 3.98-10.04 × 10³/µL). Platelet count, which was within normal limits on admission (269 × 10³/µL), decreased to 145 × 10³/µL on hospital day three (reference range 182-369 × 10³/µL). Severe hyperglycemia (serum glucose 353 mg/dL; reference range 70-99 mg/dL) and hemoglobin A1c (HbA1c) of 14% (reference range 4.0%-6.0%) indicated chronically uncontrolled diabetes mellitus. Renal function tests demonstrated severe kidney dysfunction with serum creatinine 4.31 mg/dL (reference range 0.51-0.95 mg/dL), blood urea nitrogen 52 mg/dL (reference range 7-20 mg/dL), and estimated glomerular filtration rate (GFR) of 12 mL/min/1.73 m² (reference value ≥60 mL/min/1.73 m²), findings consistent with severe renal impairment and concerning for advanced chronic kidney disease. However, prior baseline renal function was unavailable to confirm chronicity. C-reactive protein was markedly elevated at 7.97 mg/dL (reference range 0.00-0.50 mg/dL). Liver function tests showed elevated transaminases (alanine aminotransferase (ALT) 142 U/L, aspartate aminotransferase (AST) 137 U/L) and alkaline phosphatase (457 U/L). Lactic acid was at the upper limit of normal (2.2 mmol/L).

**Table 1:** Laboratory findings. ALT, alanine transaminase; AST, aspartate transaminase; ALP, alkaline phosphatase; CRP, C-reactive protein; GFR, glomerular filtration rate; BUN, blood urea nitrogen; WBC, white blood cell count

Laboratory test	Results	Reference range
WBC (K/µL)	18.85	3.98–10.04
Hemoglobin (g/L)	14.2	11.2-15.7
Hematocrit (%)	39	34.1-44.9
Platelet count (K/µL)	269	182-369
CRP (mg/dL)	7.97	0.00 - 0.50
Serum Glucose (mg/dL)	353	70-99
Hemoglobin A1c (%)	14	4.0 - 6.0
ALT (U/L)	142	5-33
AST (U/L)	137	0-32
ALP (U/L)	457	35-104
Creatinine (mg/dL)	4.31	0.51-0.95
GFR (mL/min/1.73 m²)	12	≥60
BUN (mg/dL)	52	6-20
Lactic acid (mmol/L)	2.2	0.5-2.2

Urinalysis demonstrated hematuria and evidence of urinary tract infection. Urine cultures subsequently grew Escherichia coli. Two sets of blood cultures grew Escherichia coli, confirming bacteremia, with susceptibility testing demonstrating broad antimicrobial susceptibility and no evidence of extended-spectrum beta-lactamase (ESBL) production. Viral hepatitis screening was negative, and complement levels (C3 and C4) were normal. 

**Figure 1:**
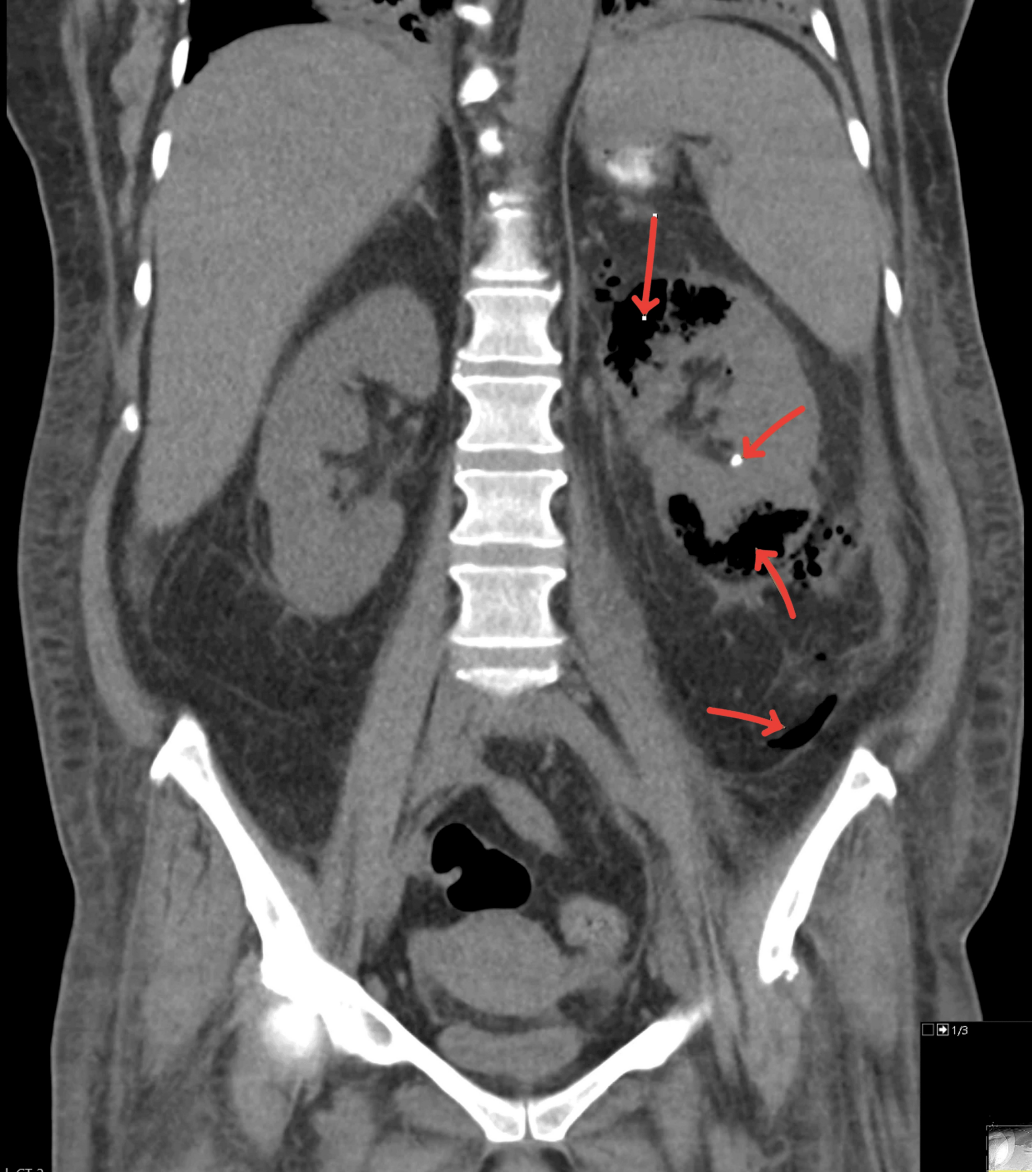
Prominent inflammatory changes and edema of the left kidney with extensive scattered free air, consistent with severe pyelonephritis. A 5 mm non-obstructing calculus is noted at the interpole of the left kidney (arrows).

Computed tomography (CT) of the abdomen demonstrated prominent inflammatory changes and edema of the left kidney with extensive scattered free air, most pronounced at the lower pole, consistent with severe EPN (Figure 1). Imaging also identified a 5-mm non-obstructing calculus at the interpolar level of the left kidney and a punctate calculus in the right kidney at a similar location. Based on radiological findings, this case was classified as Huang class 3A EPN, with gas extending to the perinephric space. 

**Figure 2:**
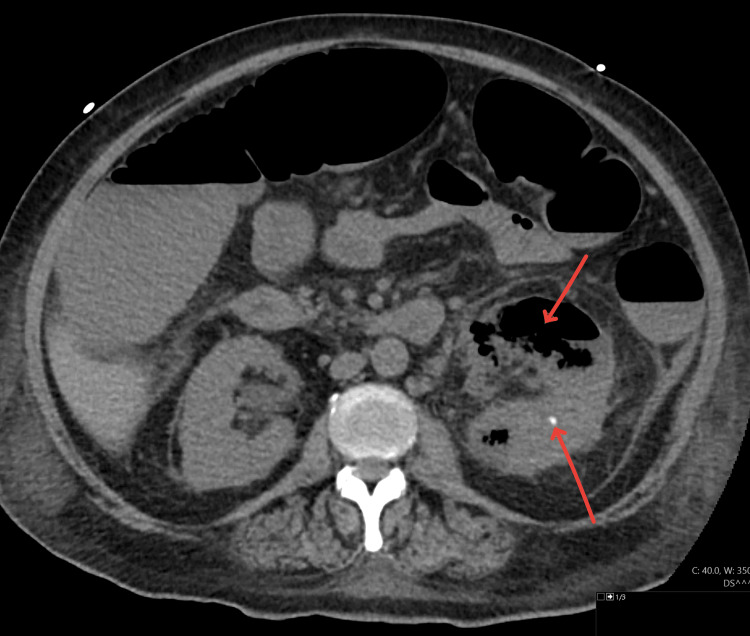
Anterior air-fluid level within the kidney, concerning for a developing intrarenal abscess (arrows). Left intrarenal calculi are redemonstrated.

A repeat CT scan performed six days after initial presentation demonstrated progression of disease with an air-fluid level within the left kidney concerning for intrarenal abscess formation, along with a small loculated fluid collection along the lateral perirenal fascia (Figure 2). This radiographic progression, despite initial therapy, indicated failure of conservative management. 

Despite prolonged antibiotic therapy and repeated drainage procedures over the subsequent week, the patient demonstrated persistent infection with ongoing clinical deterioration. The combination of newly diagnosed diabetes with extreme HbA1c elevation (14%), severe chronic kidney disease (GFR 12 mL/min/1.73 m²), bacteremia, and extensive renal involvement with abscess formation represented multiple high-risk features predictive of conservative management failure. Ultimately, the patient underwent robotic left radical nephrectomy on day 36 of illness.

**Figure 3:**
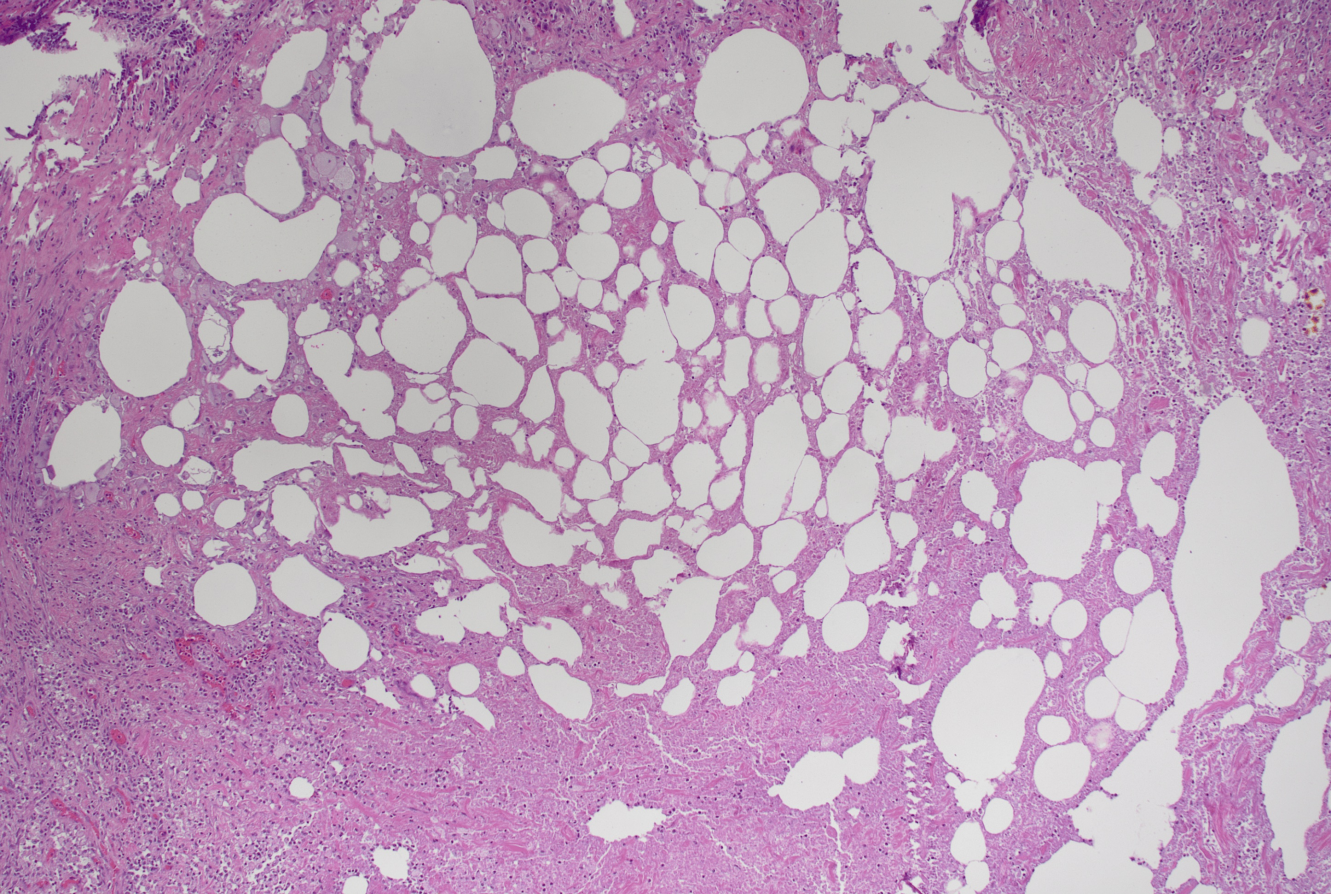
Histopathology showing areas of perinephric fat necrosis associated with severe inflammatory destruction.

**Figure 4:**
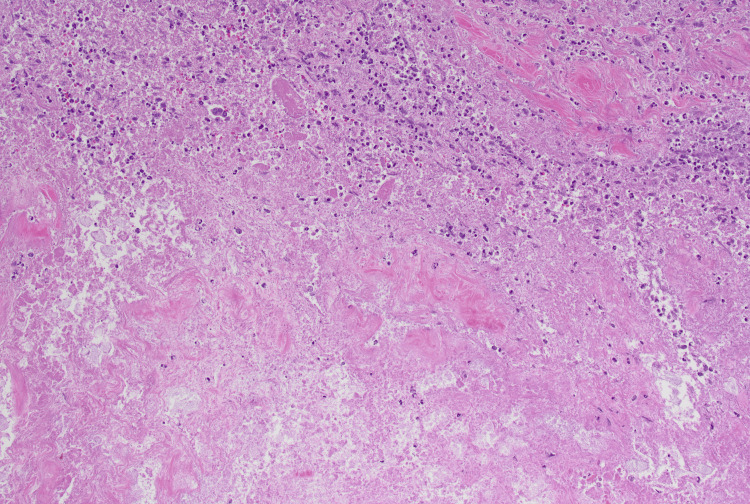
High-power image showing extensive neutrophils and abscess formation.

Gross examination of the left kidney showed extensive abscess formation, including a large lower-pole abscess measuring 5.5 cm and a smaller upper-pole abscess measuring up to 2.0 cm, with additional scattered abscesses throughout the renal parenchyma. Pathologic evaluation of the nephrectomy specimen revealed extensive abscesses with necrosis, fibrosis, dense chronic inflammation, and associated perinephric fat necrosis, consistent with severe, destructive renal infection. Conservative treatment, including ureteral stenting, failed to improve the patient’s condition, whereas surgical removal led to marked clinical recovery (Figures 3-4). 

Postoperative course was uncomplicated, with resolution of fever, normalization of inflammatory markers, and improvement in overall clinical status. She was discharged on postoperative day 5, with completion of a two-week course of oral antibiotics. At the six-week follow-up, the patient remained clinically well with complete resolution of symptoms. Renal function stabilized with the remaining right kidney, with serum creatinine improving to 0.95 mg/dL and estimated GFR increasing to 75 mL/min/1.73 m², consistent with preserved renal function and marked improvement from the initial presentation (Table 2). Diabetes management was optimized with the initiation of insulin therapy and diabetes education. HbA1c at three-month follow-up improved to 7.5%. The patient was referred to nephrology for chronic kidney disease management and endocrinology for diabetes care. She expressed understanding of the importance of regular medical follow-up and medication compliance.

**Table 2:** Key laboratory parameters at presentation and six weeks after surgery. GFR, glomerular filtration rate; WBC, white blood cell

Parameter	At presentation	Six weeks after surgery	Reference range
Creatinine (mg/dL)	4.31	0.95	0.51-0.95
GFR (mL/min/1.73 m²)	12	75	≥60
WBC (K/µL)	18.85	6.96	3.98-10.04

## Discussion

This case illustrates several important aspects of EPN management, particularly the challenges that arise when recommended treatment is interrupted and the importance of early risk stratification to identify patients who may not respond adequately to initial conservative therapy.

Our patient presented with multiple established risk factors for conservative management failure. The Huang classification system, based on radiological extent of gas and abscess formation, is the most widely used prognostic tool for EPN. Class 1 (gas in the collecting system only) and class 2 (gas in the renal parenchyma without extrarenal extension) have an excellent prognosis with medical management and percutaneous drainage, with mortality rates of 0%-18% [5]. However, class 3A (extension to perinephric space) and particularly class 3B (extension to pararenal space) carry significantly higher mortality rates of 29% and 19%, respectively, with class 4 (bilateral EPN or solitary kidney) having the worst prognosis [4,5]. Our patient's initial imaging demonstrated class 3A disease with subsequent progression to multiple abscesses. According to the Huang-Tseng classification, patients with extensive EPN (Class 3 or 4) and two or more risk factors - thrombocytopenia, acute renal failure, altered consciousness, or shock - have a 92% failure rate with percutaneous drainage combined with antibiotic therapy [5]. Thrombocytopenia is a well-established independent risk factor for mortality in EPN, with a meta-analysis demonstrating an odds ratio of 22.68 (95% CI 4.4-116.32) for mortality in patients with low platelet counts [6].

In our case, the patient presented with one definitive risk factor (acute renal failure with GFR 12 mL/min/1.73 m²). Notably, despite the severity of infection, the patient did not develop significant thrombocytopenia (nadir 145 × 10³/µL), which has been identified as a poor prognostic indicator when below 60 × 10³/µL [7]. Although the patient had only one of the four Huang-Tseng risk factors, the presence of uncontrolled diabetes (HbA1c 14%)-a well-established predisposing factor for EPN and predictor of poor outcomes-combined with failure of conservative management and recurrent abscess formation, ultimately necessitated nephrectomy. While initial percutaneous drainage was appropriate as a temporizing measure given the patient's reluctance to remain hospitalized for immediate surgery, the recurrence of abscess at Day 22 represented definitive failure of conservative management and should have prompted immediate nephrectomy rather than repeat drainage [8].

A recent study on surgical pyelonephritis found that 11.5% of patients initially treated conservatively required secondary nephrectomy, with diabetes, chronic kidney disease, and high ASA scores identified as predictors of failure [9]. The eventual 36-day delay to definitive surgical management likely contributed to prolonged morbidity. The transition to oral amoxicillin-clavulanate was inappropriate, not because of insufficient IV antibiotic duration (12 days falls within the standard 10- to 14-day range for complicated UTI), but because source control had not been achieved [10]. Repeat imaging on day 6 demonstrated abscess progression with air-fluid levels, and percutaneous drainage had only been performed one day before discharge. Oral step-down therapy is appropriate only when the patient demonstrates clinical improvement and source control has been established. In this case, the patient's premature self-discharge prevented adequate monitoring of treatment response, including normalization of inflammatory markers and confirmation of abscess resolution on follow-up imaging. The patient's profound immunocompromise, with uncontrolled diabetes (HbA1c 14%), further heightened the risk of treatment failure, as impaired polymorphonuclear leukocyte function and defective phagocytosis in hyperglycemic states reduce the host's ability to clear residual infection, even with appropriate antimicrobial coverage [4]. The combination of an uncontrolled infectious source, lack of confirmed clinical improvement, and severe immunocompromise likely contributed to the abscess recurrence. This treatment failure underscores the importance of timely surgical escalation when conservative management proves inadequate in high-risk patients.

In a study of 112 patients with EPN, approximately 60% improved with percutaneous drainage alone; however, a subset of patients managed with medical therapy and drainage ultimately required nephrectomy [11]. The literature demonstrates that patients with extensive disease, poor glycemic control, and failure to improve within 72 hours of drainage often require surgical intervention [5]. The patient's discharge on day 12 against the urology team's recommendation for nephrectomy created a gap in care that allowed disease progression. Repeat imaging upon readmission revealed a second abscess that might have been prevented with timely surgical intervention. The chronic inflammatory milieu in this patient likely contributed to the extensive tissue destruction observed on final pathology, with multiple abscesses, necrosis, fibrosis, and perinephric fat involvement confirming that the kidney was unsalvageable by the time nephrectomy was performed.

This case illustrates how patient reluctance to undergo recommended surgery can alter the trajectory of severe infections. Strategies to prevent premature discharge in life-threatening cases include explicit communication of mortality risk (reported up to 50% in untreated extensive EPN) [2], involvement of family members to reinforce the urgency of treatment, and multidisciplinary meetings to address barriers to hospitalization. Patients who leave against medical advice have a two- to fourfold higher readmission rate and increased morbidity and mortality. Motivational interviewing, establishing trust through active listening and empathy, and negotiating treatment plans that afford patients some sense of autonomy may help prevent premature discharge in such cases [12]. This case makes a valuable contribution to the literature by documenting the consequences of treatment interruption in EPN, a real-world challenge that is underreported in existing case series, which typically describe patients who complete recommended therapy.

## Conclusions

This case highlights the importance of early recognition of emphysematous pyelonephritis in patients with poorly controlled diabetes mellitus and severe systemic illness. Extensive gas formation and abscess development may indicate advanced disease requiring prompt escalation of care. Early risk stratification and timely source control, including percutaneous drainage or nephrectomy when clinically indicated, are critical to preventing disease progression and improving outcomes. This case also underscores that prolonged antimicrobial therapy alone may be insufficient when adequate source control has not been achieved.
